# Metabolic targeting, immunotherapy and radiation in locally advanced non-small cell lung cancer: Where do we go from here?

**DOI:** 10.3389/fonc.2022.1016217

**Published:** 2022-12-14

**Authors:** Annika Dhawan, Phillip M. Pifer, Vlad C. Sandulache, Heath D. Skinner

**Affiliations:** ^1^ Department of Radiation Oncology, UPMC Hillman Cancer Center and University of Pittsburgh, Pittsburgh, PA, United States; ^2^ Bobby R. Alford Department of Otolaryngology-Head and Neck Surgery, Baylor College of Medicine, Houston, TX, United States

**Keywords:** lung cancer, radiation, chemo-radiotherapy, metabolism, metformin, immunotherapy

## Abstract

In the US, there are ~250,000 new lung cancer diagnoses and ~130,000 deaths per year, and worldwide there are an estimated 1.6 million deaths per year from this deadly disease. Lung cancer is the most common cause of cancer death worldwide, and it accounts for roughly a quarter of all cancer deaths in the US. Non-small cell lung cancer (NSCLC) represents 80-85% of these cases. Due to an enormous tobacco cessation effort, NSCLC rates in the US are decreasing, and the implementation of lung cancer screening guidelines and other programs have resulted in a higher percentage of patients presenting with potentially curable locoregional disease, instead of distant disease. Exciting developments in molecular targeted therapy and immunotherapy have resulted in dramatic improvement in patients’ survival, in combination with new surgical, pathological, radiographical, and radiation techniques. Concurrent platinum-based doublet chemoradiation therapy followed by immunotherapy has set the benchmark for survival in these patients. However, despite these advances, ~50% of patients diagnosed with locally advanced NSCLC (LA-NSCLC) survive long-term. In patients with local and/or locoregional disease, chemoradiation is a critical component of curative therapy. However, there remains a significant clinical gap in improving the efficacy of this combined therapy, and the development of non-overlapping treatment approaches to improve treatment outcomes is needed. One potential promising avenue of research is targeting cancer metabolism. In this review, we will initially provide a brief general overview of tumor metabolism as it relates to therapeutic targeting. We will then focus on the intersection of metabolism on both oxidative stress and anti-tumor immunity. This will be followed by discussion of both tumor- and patient-specific opportunities for metabolic targeting in NSCLC. We will then conclude with a discussion of additional agents currently in development that may be advantageous to combine with chemo-immuno-radiation in NSCLC.

## Introduction

In the US, there are ~250,000 new lung cancer diagnoses and ~130,000 deaths per year, and worldwide there are an estimated 1.6 million deaths per year from this disease. Lung cancer is the most common cause of cancer death worldwide, and it accounts for roughly a quarter of all cancer deaths in the US (1, 2). Non-small cell lung cancer (NSCLC) represents 80-85% of these cases. Due to an enormous tobacco cessation effort, NSCLC rates in the US are decreasing, and the implementation of lung cancer screening guidelines and other programs have resulted in a higher percentage of patients presenting with potentially curable locoregional disease, instead of distant disease (3). Exciting developments in molecular targeted therapy and immunotherapy have resulted in dramatic improvement in patients’ survival, in combination with new surgical, pathological, radiographical, and radiation techniques. Concurrent platinum-base doublet chemoradiation therapy followed by immunotherapy has set the benchmark for survival in these patients (4). However, despite these advances, ~50% of patients diagnosed with locally advanced NSCLC (LA-NSCLC) survive long-term (4). In patients with local and/or locoregional disease, chemoradiation is a critical component of curative therapy. However, there remains a significant clinical gap in improving the efficacy of this combined approach, and the development of non-overlapping treatment approaches to improve treatment outcomes is needed.

One potential promising avenue of research is targeting cancer metabolism. In this review, we will initially provide a brief general overview of tumor metabolism as it relates to therapeutic targeting. We will then focus on the intersection of metabolism on both oxidative stress and anti-tumor immunity. This will be followed by discussion of both tumor- and patient-specific opportunities for metabolic targeting in NSCLC. We will then conclude with a discussion of additional agents currently in development that may be advantageous to combine with chemo-immuno-radiation in NSCLC.

## Metabolic shifts during cancer development uncover therapeutic vulnerabilities

As first detailed by Otto Warburg nearly a century ago, metabolic activity within tumor cells diverges significantly from normal eukaryotic homeostasis (5). Whereas normal cells balance energy and biomass to support specialized functions and proliferation, tumor cells disproportionately favor high proliferative activity. This results in a metabolic program designed to generate high biomass turnover at the expense of balanced energy production (6). Common metabolic pathways including glycolysis, pentose phosphate, tricarboxylic acid (TCA) cycle, and oxidative phosphorylation are all affected, and their activity is coordinated to support enhanced proliferation even at the expense of energetic efficiency. Intermittent and variable hypoxia, which is ubiquitous in solid tumors, further exacerbates this phenotype.

Cancer cells frequently encounter hypoxic conditions which result in mitochondrial membrane hyperpolarization, leading to inhibition of mitochondrial transition pores that normally release pro-apoptotic factors. In these hypoxic conditions, hypoxia-induced factor 1-alpha (HIF1α) is activated, which increases glucose transporter expression. This allows increased glucose into the cell, amplifies transcription of glycolytic enzymes, and prompts a switch to aerobic glycolysis to produce ATP (7, 8). HIF1α activates pyruvate dehydrogenase kinase 1 (PDK1) which inhibits pyruvate dehydrogenase (PDH), and therefore reduces the supply of acetyl-CoA entering the TCA cycle, shunting carbon flux elsewhere (7). This mitochondrial dysregulation, in both normoxic and hypoxic conditions, allows cancer cells to use excess pyruvate for anabolic synthesis (7). Cancer cells additionally show increased glutamine uptake for glutaminolysis, which refills TCA intermediates that are shunted into heightened biosynthetic processes (9).

Cancer cells also undergo metabolic shifts during treatment with radiation and chemotherapy (10). Radiation’s therapeutic effect is the result of unrepaired DNA damage leading to cell death, and cells must use DNA damage repair pathways to avoid death. DNA damage repair is an energy intensive process, and cancer cells already have a high energy demand making it difficult to allocate excess energy for this process (11, 12). Radiation also induces PI3K and NF-kB pathways along with epithelial-mesenchymal transition transcription factors (SNAI1, HIF1, ZEB1, and STAT3) which can result in metabolic reprograming (11, 13, 14). The effect of chemotherapies on metabolism is vast, depends upon the agent in question, and has been extensively reviewed previously (8, 15). Different chemotherapies can affect glycolysis, fatty acid synthases, glutaminolysis, and other metabolic pathways (15). Chemotherapies promote metabolism shifts that result in drug resistance (16).

Although well designed for maximal proliferation, these metabolic adaptations toward higher biomass turnover present interesting opportunities for therapeutic intervention. First, it is important to note that under conditions of stress such as that generated by chemoradiation, the biomass requirements for survival may surpass even those of normal cancer proliferation and thus present an opportunity for effective combinatorial strategies. Second, targeting of biomass-generating pathways may be preferentially effective in cancer cells while limiting toxicity in normal tissue. It is important to remember that metabolic pathways which are partially dysregulated can become severely sensitive to disruption even with limited inhibition. As such, a third opportunity arises from minimal inhibition of a generally altered pathway (e.g., oxidative phosphorylation) to generate disproportionate reductions in energy and biomass resulting in catastrophic tumor cell death.

## Metabolic regulation of oxidative stress

How tumor cells regulate intra-cellular ROS remains unclear, but it appears to require a careful interplay between intrinsic tumor biology and the tumor microenvironment (TME). The TME includes the surrounding host immune cells, extracellular matrix (ECM), blood vessels, fibroblasts, lymphocytes, other bone marrow-derived inflammatory cells, and signaling molecules (17). Often, in solid tumors, the TME is characterized by hypoxia, low pH, and high interstitial fluid pressure (18). These conditions further the shift from oxidative respiration to glycolysis and increase lactic acid production resulting in an acidic TME (19). This acidosis boosts reactive oxygen species (ROS) formations leading to RAS-RAF-MEK-MAPK pathway activation, thereby promoting cell proliferation (19, 20).

However, free radicals are a double-edged sword in the context of solid tumors. On the one hand, tumor cells–particularly aggressive, highly proliferative and metastatic cells–appear to thrive in the presence of slightly elevated ROS levels, suggesting that higher ROS levels may be pro-tumorigenic (21). On the other hand, conventional chemotherapy and radiation generate much of their anti-tumorigenic activity by dramatically increasing ROS levels above those which tumor cells can absorb without catastrophic damage to their DNA (22). The TME is a site of significant interaction between these cancer therapeutics and cancer metabolism, and the TME can promote cancer survival by impairing the efficacy of radiation, chemotherapy, and immunotherapy (18, 23–25). Since the cytotoxic effects of radiation are potentiated by oxygen, hypoxic tumor stromal regions of the TME can drive radioresistance and local treatment failure (18). The acidification of the TME promotes cancer cell metabolic reprogramming, selects for cancer cells that are resistant to hypoxic conditions, and therefore promotes chemoresistance and metastatic development (25). The TME and ROS production present a targetable area for cancer therapeutics that will be discussed further in this review.

## Metabolic effects on anti-tumor immunity

Even in the absence of immunotherapy, a functional immune system is important for maximal response to treatment in solid tumors, including NSCLC (26). In patients with NSCLC, the presence of tumor-infiltrating lymphocytes (TILs) is associated with improved distant-free survival and overall survival (27–30). Radiation improves antitumoral immunity in the TME by increasing the release of tumor antigens and allowing antigen-presenting cells to activate CD8+ T-cells. This ultimately results in tumor cell death (31, 32).

As demonstrated in the PACIFIC trial, the benefit of adjuvant PD-L1 blockade using durvalumab after chemoradiation in NSCLC is thought to be at least partially due to its ability to block inhibition of CD8+ cytotoxic T cells, which are presumably activated following chemoradiation. The interplay between a broadly immunosuppressive therapy – namely chemoradiation – with these immunogenic characteristics leads to a complicated and not completely understood interaction. The timing of immune-checkpoint blockade is critical as initial studies in other cancers that used immune-checkpoint blockade concurrently with chemoradiation have underperformed (33), unlike in the consolidation setting of immune checkpoint blockade in PACIFIC.

Adding to this complexity is the effect of tumor metabolism on the TME. In the TME, excess lactate is immunosuppressive (25, 34). The tumor stroma suppresses host immune responses against malignant tumor cells: restricting T-cells from making contact with the cancer cells, secreting immunosuppressive cytokines, and metabolic competition between tumor cells and immune cells (32). Members of the host immune system such as B-cells, T-cells, NK cells, dendritic cells, and macrophages are present in the TME, yet the interactions between malignant and non-malignant cells in the TME create a carcinogenic environment with metabolic stress and often render immune cells less functional (24). The TME induces host immune cells to become tolerogenic to cancer cells, and the immune cells become unproductive in cancer suppression. This ultimately results in decreased immunotherapy effectiveness (35).

Drugs targeting cancer metabolism may synergistically enhance immunotherapy due to the significant overlap with the TME (36). Indeed, agents targeting tumor metabolism could have multiple benefits on immune response both by creating a TME more favorable to immune function as well as directly affecting the tumor-infiltrating lymphocytes to improve their cytotoxic function (37). Some examples of metabolic intervention tactics to improve immunotherapy response include: 1) PI3K-AKT-mTOR blockade which can simultaneously inhibit cancer metabolism and disinhibit the immune system *via* inhibition of regulatory T cells (38) and 2) targeting glutaminolysis *via* glutaminase (GLS) or arginase 1 to promote differentiation and function of CD4 and CD8 T cells (39–42). In an in-depth analysis, Guerra et al. argue that different players in the TME use or modify energetic pathways in different types of cancers which could be used to design cancer-specific immune metabolic modulators. These approaches in metabolic manipulation in combination with chemoradiation may enhance immune cell fitness in the TME. Specifically, because radio- and chemotherapy induce tumor cell death, more nutrients become available in the TME for immune cells to use to their own advantage (35).

The complexity of the immune system with multiple immune cell types using different metabolic pathways and responding to different stimuli should not be understated (43). Metabolic manipulation must be done in a thoughtful way to promote anti-tumor immunity and not blunt its response (14, 44). For example, once stimulated, effector T cells increase glycolysis and macronutrient production (45), but memory T cells rely on OXPHOS with a lower glycolysis rate (45, 46). Tumor cells have also been demonstrated to competitively uptake glucose to inhibit TILs (47), whereas PD-1 blockade increases glucose in TILs and promotes glycolysis (35). An in-depth review of different immune cell metabolic profiles has been previously published (48). These findings suggest caution when using metabolic modulation anti-cancer therapies in combination with immunotherapy, and that temporal and spatial interaction will be important.

As noted previously, cancer’s metabolic adaptations to the tumor microenvironment create barriers to effective immunotherapy applications, supporting the addition of metabolic modulators to therapeutic regimens for NSCLC (34). Metabolic modulator use in the treatment of NSCLC may improve radiotherapy and immunotherapy efficacy, and the interplay between therapeutic agents in the TME supports the continued exploration of chemoradiation, immunotherapy, and metabolic modulators.

## Opportunities for metabolic targeting in lung cancer

Completion of TCGA-led efforts over the last decade have unraveled the genomic, transcriptomic, and proteomic landscape of lung cancer in a manner never previously thought possible (49, 50). Among NSCLC subtypes, adenocarcinomas have a higher incidence of actionable mutations and are more prevalent in non-smokers (50). Squamous cell carcinomas have a higher frequency of non-actionable mutations such as *TP53* (~80-90% of cases) and are more common in smokers (49, 51). The two most commonly mutated genes in NSCLC are *TP53* and *KRAS*, with *KRAS* being almost exclusively in adenocarcinomas. The two most commonly mutated signaling pathways are PI3K-Akt-mTOR and RAF-MEK-MAPK (50). By examining these mutations, potential candidates for metabolic modulation can be identified.

*TP53* is mutated in 46% of NSCLC tumors (50), and mutated *TP53* is associated with worse patient outcomes (52). Although not directly actionable currently, *TP53* mutations result in loss of normal protein function which provide several unique opportunities for metabolic intervention. First, mutated *TP53* results in reduced apoptosis by *TP53-induced glycolysis and apoptosis regulator* (TIGAR) suppression and increased glycolysis and glucose transport by expression of phosphoglucomutase (PGM), losing the ability to block G6PD activity (53). Second, when glycolysis is pharmacologically inhibited, mutant *TP53* NSCLC tumor cells lines are unable to upregulate OXPHOS compared to wild type *TP53*, suggesting a mechanism to target mutant *TP53 (* 54). The high prevalence of *TP53* mutations makes it an attractive target in NSCLC.

The RAS-RAF-MEK-MAPK pathway is mutated in 58% of all NSCLC tumors and 76% of lung adenocarcinomas (50, 55). In lung adenocarcinoma, activating mutations in oncogenes *KRAS* (32%), *EGFR* (11%), and *BRAF* (7%) are common (50). It should be noted that *KRAS* and *EGFR* mutations are mutually exclusive. Alteration in this pathway results in heightened expression of central glycolytic enzymes. Oncogenic *RAS* mutations keep RAS membrane proteins in their GTP-bound, active state, resulting in uncontrolled growth (8). *KRAS* mutations have higher prevalence in smokers, but are still seen in non-smokers (56). Mutated *KRAS* is associated with radioresistance in NSCLC and other cancers (57–59). Mutated *KRAS* NSCLC tumors are associated with higher expression of PD-L1/2, promoting an immunoevasive environment (60). Once thought to be untargetable, a newly approved *KRAS* small-molecular inhibitor for patients with *KRAS* ^C12C^ demonstrated an objective response rate of ~33%, and provides hope of being able to improve outcomes in these patients (61). Mutated *KRAS* affects reactive oxygen species (ROS) regulation and promotes radio- and chemoresistance (59, 62).

The PI3K-Akt-mTOR signal transduction pathway manages glucose regulation and activates oncogenic mutations leading to angiogenesis, proliferation, and differentiation among other downstream effects (8). Therefore, it provides a direct link between tumorigenesis, treatment response, and metabolic targeting. In NSCLC, common mutations of this pathway include serine/threonine kinase 11 (*STK11* aka *LKB1*) which is inactivated in 17% of cases, *PTEN* (3%), *PIK3CA* (4%), and *AKT1* (1%) (50). *STK11* encodes the tumor suppressor enzyme serine/threonine kinase 11, which when lost can increase flux of glucose-derived carbon towards serine biosynthesis, support DNA methylation, and promote oncogenic metabolic phenotypes (63). Loss of *STK11* is associated with locoregional recurrence after radiation (64).The mechanism of resistance is in part driven by the Kelch-like ECH-associated protein 1 (*Keap1*)/nuclear factor erythroid-2-related factor 2 (*NFR2*) pathway (64, 65).

The *Keap1/NRF2* pathway is essential in metabolic and ROS regulation. In homeostatic conditions, Keap1 with other proteins ubiquitinates Nrf2 leading to its degradation *via* proteosomes (66). When cells undergo an oxidative stress, Keap1 is oxidized preventing Nrf2 ubiquitination which results in Nrf2 accumulation (67). Nrf2 is able to translocate to the nucleus, interacts with nuclear proteins, and transcriptionally activates antioxidant gene response (68). Nrf2 regulates Mdm2, NAD(P)H quinone dehydrogenase 1 (NQO1), heme oxygenase 1 (HMOX1), and glutamate-cysteine ligase modifier subunit (GCLM) among others (69–72). As an oxidative stress regulator, Nrf2 is important in the regulation of cancer metabolism. It up-regulates metabolic pathways including glycolysis/gluconeogenesis, pyruvate metabolism, pentose phosphate pathway, glutathione metabolism, and others leads to increased production of catabolic building blocks of nucleic acids (73), carbohydrates, amino acids, and lipids which are vital for tumor survival (65). Because KEAP1 is inactivated by high levels of ROS resulting in activation of Nrf2, Nrf2 is one of the master regulators of anti-oxidative transcription factors.

In NSCLC, loss-of-function mutations in *Keap1* and gain-of-functions of *Nrf2* have been observed in 11.3% and 3.5% of patients, respectively (74). These mutations result in a constitutively active Nrf2 (75, 76). In patients with lung adenocarcinoma, patients with high Keap1 and low Nrf2 have better clinical outcomes than patients with low Keap1 and high Nrf2 (74, 76). In NSCLC, mutated *KRAS* leads to *NRF2* upregulation promoting chemoresistance and radioresistance (62, 77). Nrf-2 inhibition has been demonstrated to be a radiosensitizer in other cancers as well (78). Co-mutation of *KRAS* and *KEAP1/NFE2L2* predicts worse survival to chemotherapy and immunotherapy (79), and Nrf-2-regulated ROS homeostasis has been linked to chemotherapy resistance in NSCLC and other cancers (76, 80–83). Knockdown of NRF2 in NSCLC cell lines results in higher basal ROS causing radiosensitization (84). When examining subtype-specific transcriptional phenotypes in both *KRAS* mutant and wild type NSCLC, the proliferative tumor subtype were *STK11/KEAP1* deficient in ~90% and ~75% of *KRAS* mutant and wild type tumors, respectively (85), and the proliferative tumor subtype appeared to respond to MEK inhibition (85). The small molecule inhibitor of Nrf-2, IM3829, resulted in radiosensitization in *in vitro and in vivo* experiments in NSCLC and demonstrated expected increase in ROS and apoptosis (86). Due to Nrf-2 promoting both radioresistance and chemoresistance, NRF-2 inhibition makes for a rational target for improving NSCLC chemoradiation. Targeting the KEAP1/NFR2 pathway by inhibiting glutaminase may radiosensitize tumors with the *STK11* mutation (64). Glutaminase (GLS) inhibition blocks the conversion of glutamine to glutamate, decreasing glutathione production. Inhibition of GLS by CB-938 leads to NSCLC tumor cell sensitization to radiation (87–89). GLS inhibitors are currently in clinical trials in solid tumors with chemotherapy combination, but not currently any with radiation (90, 91). This example demonstrates the potential for metabolic modulators to improve concurrent chemoradiation.

Moreover, bioinformatic analysis of multiple smoking related malignancies including NSCLC has identified a strong correlation between Nrf2 hyperactivation and a suppressive tumor immune microenvironment, depleted of effector immunocytes (Sandulache et al., In press). Experimental data both *in vitro* and in immunocompetent pre-clinical models have identified glutathione peroxidase 2 (GPX2), an Nrf2 target and a critical regulator of oxidative stress response, as a primary driver of suppressed immunity, manifested through reduced cytokine and chemokine production by tumor cells, development of tumors depleted of cytotoxic T cells, and enriched for myeloid derived suppressor cells.

AMP-activated protein kinase (AMPK) is a key regulator of cellular energy metabolism implicated in the PI3K-Akt-mTOR cell signaling pathway, an important molecule in both metabolic and genomic signaling pathways (92–94), and induces p53 activation promoting cell survival and senescence (95–97). AMPK is targeted in metabolic syndrome and type 2 diabetes mellitus, and it has been proposed as a metabolic tumor suppressor in cancer treatment (98). In cancer microenvironments, insulin activates the PI3K/Akt signal transduction pathway and inhibits GSK3, a tumor suppressor that inactivates glycogen synthase by phosphorylation (99, 100). GSK3 inhibition results in cancer cells storing and synthesizing glycogen in abnormally high quantities, promoting cancer cell survival (101). SKT11 (aka LKB1) is upstream of AMPK, normally leading to its activation, however this effect may not be present in the setting of *STK11* mutation (102). As radiation normally activates AMPK, and reversal of this activation leads to radioresistance, this may be a mechanism by which *STK11* mutation drives therapeutic response independent of *Keap1/Nrf2* (92, 93).

The high rates of mutations of *TP53* and *KRAS* and dysregulation of PI3K-Akt-mTOR and RAF-MEK-MAPK mechanistically supports and demonstrates the importance of enhanced biomass requirements of rapidly proliferating cells. This is particularly seen in conditions of energy deprivation seen in the TME (e.g., AMPK sensing low ATP levels). Although aberrant in cancer, NSCLC must tightly regulate the metabolism, otherwise, the most likely outcome is a catastrophic metabolic disruption resulting in cell death. By exploiting this careful balance, metabolic modulators may enhance the effects of radiation, chemotherapy, and immunotherapy.

## Systemic metabolic dysregulation and NSCLC

Metabolic dysfunction is a risk factor for many types of cancer. Hyperinsulinemia and hyperglycemia predict for increased cancer incidence and worse patient survival outcomes in a variety of cancers (103, 104). Diabetic cancer patients experience worse prognoses than non-diabetic patients, resulting in a 0-40% increase in overall mortality for different types of cancer (105). Diabetes mellitus is an independent prognostic factor for worse outcomes in patients with locally advanced NSCLC (106), and hyperglycemia predicted for worse survival in these patients when treated with concurrent chemoradiation (107). Paradoxically, obesity is linked with decreased risk of lung cancer and better outcomes. This has been attributed to confounders such as smoking and lung cancer-associated weight loss (108–110). In metabolic syndrome (characterized by three of five characteristics of abdominal obesity, hypertriglyceridemia, hyperlipidemia, hypertension and/or hyperglycemia), there has also been association with increased risk of certain cancers (111). Both obesity and metabolic dysfunction are associated with a state of chronic inflammation (111, 112). Emerging evidence demonstrates that obesity creates an immunosuppressive environment in the TME (113), but there is evidence demonstrating patients with obesity have better responses to immunotherapy, especially in NSCLC (114). Because of these links between metabolic dysregulation and cancer, investigation into effective metabolic modulators is warranted to potentially incorporate them into cancer therapeutics and understand the full implications between these two diseases.

In patients with type 2 diabetes, metformin is considered the initial drug of choice for management of hyperglycemia (115). Metformin inhibits hepatic gluconeogenesis *via* interaction with the mitochondrial electron transport chain and disrupts cAMP-PKA signaling (116). Epidemiologic studies in diabetic patients show that metformin use is associated with decreased risk of cancer in comparison to other anti-diabetic treatments (117), and specifically in NSCLC incidence (118–120). In diabetic patients taking metformin there is a survival benefit compared to diabetic patients not taking metformin or the nondiabetic population in all cancers (121). In other solid cancer subsites, metformin use is associated with improved outcomes with radiotherapy and chemoradiotherapy (122–124). In advanced NSCLC, patients with type II diabetes taking metformin had improved PFS and OS compared to those patients taking insulin or other anti-diabetic medications (125). In patients with diabetes and non-operable NSCLC, metformin use is associated with significantly longer overall survival suggesting that metformin may have an anti-tumorigenic effect (126).

In preclinical models, metformin suppresses oncogenic pathways such as EGFR signaling, insulin-like growth factor, and acts as a radiation sensitizer in NSCLC by activating AMPK, suppressing mTOR, and inducing G1 cell-cycle arrest (98, 127–129). Metformin inhibits NSCLC cancer growth and increases radiosensitivity with limited effect on normal lung cells (128). The mechanism of action of metformin is thought to be inhibition of the mitochondrial complex I of oxidative phosphorylation *via* mitochondrial complex I (130). Phenformin, a mitochondrial inhibitor and analog of metformin, has been shown to selectively induce apoptosis in *STK11*-deficient NSCLC cells and prolong survival in murine models of NSCLC tumors with *KRAS* and *STK11* mutations (131).

Metformin also demonstrates potentiation of antitumor immunity. In a preclinical model of head and neck cancer, long-term metformin treatment reduces tumor growth velocity, increases tumor infiltrating lymphocyte levels, and increases the CD8+/T-reg ratio (a measure of immune cell upregulation) thereby creating local and systemic immune effects (132). Furthermore, given that hypoxia in the TME acts as a barrier to immunotherapy, a recent study determined that metformin led to reduction of tumor hypoxia which improved the efficacy of PD-1 blockade and could reduce immunotherapy resistance (133).

Based on this promising preclinical work and retrospective clinical data, metformin has been pursued as a therapeutic sensitizing agent. Metformin is one of the top ten prescribed pharmaceuticals in the US, with a favorable adverse effects profile. Metformin is associated with low rates of hypoglycemia and is generally well-tolerated, with most common side effects being gastrointestinal. While rare, lactic acidosis is the most severe side effect of metformin use as a diabetic treatment. This safety and side effect profile made metformin an optimal target for incorporation with chemoradiation in NSCLC clinical trials in non-diabetic NSCLC patients and its potential synergy with immunotherapy.

Unfortunately, the integration of metformin into clinical trials for cancers has underperformed. In the locally advanced NSCLC definitive chemoradiation setting, recent clinical trials demonstrate no benefit of metformin use. NRG-LU001, a phase II clinical trial of non-diabetic patients with locally advanced unresectable NSCLC, tested the addition of metformin to concurrent chemoradiation. It demonstrated no significant difference in PFS or OS at one year compared to standard chemoradiation, failing to meet its primary endpoint (134, 135). The OCOG-ALMERA trial, a phase II clinical trial in non-diabetic patients with unresectable locally advanced NSCLC, demonstrated that concurrent and adjuvant metformin with chemoradiation compared to chemoradiation alone had worse 1-year treatment failure, 69.2% versus 42.9%, p =0.05, and 1-year PFS, 34.8% versus 63.0%, respectively (136). There were also higher rates of grade 3+ adverse events in the metformin arm (136). These trials demonstrate that metformin does not improve outcomes with standard of care chemoradiation in an unselected non-diabetic patient population.

A phase II clinical trial in inoperable early-stage NSCLC tested the addition of neoadjuvant and concurrent metformin to hypofractionated radiation (50 Gy in 4 fractions or 70 Gy in 10 fractions). Metformin resulted in increased SUV in tumors at mid-treatment PET after neoadjuvant metformin use, contrary to its expected effect on most physiologic tissues. Metabolic responses determined with PERCIST criteria on PET imaging show complete metabolic response in 69% of the metformin cohort 6 months post-radiation treatment (137). In a Phase II trial of non-diabetic stage IIIB/IV patients with *EGFR*-mutated lung adenocarcinoma, the addition of metformin to *EGFR* tyrosine kinase resulted in improved PFS and OS (138). These results offer hope for properly selected NSCLC patient populations such as those patients with *EGFR* or *KRAS/STK11* mutations (139).

Metformin became an attractive target for investigation in the clinical setting for two reasons. First, the preclinical data in multiple disease models was fairly compelling both in the single agent setting and when combined with chemoradiation (98, 127–129). Second, metformin has an unparalleled safety profile as a systemic agent used for DM management. This made it perfect for repurposing. Unfortunately, metformin failed to benefit an unselected patient population in combination with chemoradiation in NSCLC. This highlights the complexity of interactions between radiation, chemotherapy, cancer metabolism, and the TME. However, results such as those in *EGFR*-mutated lung adenocarcinoma gives hope that in an appropriately selected patient population, metformin could improve outcomes.

## Novel metabolic modulators, targets, and strategies

Most strategies rely on inhibition of major metabolic pathways, predominantly glycolysis with the exception of the *Nrf2* based strategies outlined above. This poses substantial challenges as they relate to both efficacy and normal tissue toxicity. Multiple potential metabolic enzyme-targeting therapies currently in preclinical and clinical trials not involving NSCLC are shown in **Table 1**. Those being tested in NSCLC are discussed here and summarized in **Table 2**. Future exploration focuses on inhibition of glucose transport and metabolism and inhibition of monocarboxylate transport to prevent lactate excretion.

**Table 1:** Inhibitors of metabolic enzymes in (pre)clinical development among various cancer types.

Drug	Target	Cancer Type	Status
AG-120 (ivosidenib) IDH305 BAY1436032	Mutant IDH 1/2Mutant IDH 1/2Mutant IDH 1/2	Glioma and Leukemia	Preclinical (140–143)
AZD3965	MCT1	B cell lymphoma models	Phase 1 clinical trial NCT01791595 (144, 145)
TVB-2640	Fatty acid synthase	Breast cancer	Preclinical (146)
IACS-010759	Mitochondrial respiratory complex I	*In-vitro* hypoxic tumor cells	Preclinical (147)
Dichloroacetate	PDK	Variety of cancers (NSCLC, breast cancer, glioblastoma)	Orally available for metabolic conditions (7)
Lonidamine	HK	Breast cancer	Hepatic toxicity in Phase III human trials (8, 148)

**Table 2:** Inhibitors of metabolic enzymes in preclinical/clinical development for NSCLC.

Drug	Target	Status
WZB117 DRB18 Fasentin	GLUTGLUTGLUT	Preclinical (149–151)
Shikonin	PKM2	Response in late-stage NSCLC (152, 153)
Dichloroacetate (DCA)	PDK	Preclinical development for NSCLC. Safety concerns in clinical trials (154, 155).
PSTMB	LDHA	Preclinical (8, 156)

Glucose transporters (GLUT) inhibitors (fasentin, phloretin, STF-31, DRB18 and WZB117) are in preclinical development for lung cancer (8). WZB117 and DRB18 demonstrated *in vitro* and *in vivo* inhibition in NSCLC cancer models (149, 150). Pyruvate kinase M2 (PKM2) activation results in a higher ratio of glycolysis to glucose oxidation and appears to be inactivated in cancer (7). Shikonin, a PMK2 inhibitor, demonstrated response rates in late-stage lung cancer in patients who were not candidates for surgery, radiotherapy, or chemotherapy (152, 153). Pyruvate dehydrogenase kinase 1 (PDK1) blocks PDH activity. Dichloroacetate (DCA), a PDK inhibitor, is an orally available drug that shows potential benefit in many cancer types, including reduced tumor growth, increased apoptosis *in vivo*, and a shift from glycolysis to OXPHOS in NSCLC 7 (**Table 1**) (154). However, a phase II clinical trial in metastatic NSCLC or breast cancer investigating DCA response demonstrated safety and risk concerns and was stopped prematurely (8). Lactate dehydrogenase A (LDHA), which converts cytosolic pyruvate into lactate (157), has been associated with radioresistance and chemoresistance in other cancers (158, 158). LDHA is clinically targetable by PSTMB, which demonstrates induction of apoptosis in NSCLC cancer cell lines (8). It has been proposed that LDHA inhibition must be done with careful dose and temporal control due to the significant off-target side effects of this molecule (159). Due to the importance of these metabolic proteins, this might apply to a substantial number of metabolic inhibitors and possibly limit the translation to the clinic. Another potential metabolic target is fructose-1,6-bisphosphatase (FBP1), which when aberrant causes dysfunction in NK cells of the immune system. Inhibition of FBP1 suggests restoration of NK cell function during tumor growth (160). However, it remains unclear whether these approaches can achieve significant efficacy without normal tissue toxicity.

## Metabolic imaging and treatment modulation

Although most of this discussion has focused on the addition of potential metabolic modulators to concurrent chemoradiation, the use of metabolic imaging is vital for target delineation of current radiation therapy. Molecular imaging in lung cancer has been discussed extensively in these previous reviews (161–163). Fluorodeoxyglucose (FDG)-PET scans are standard of care for the appropriate staging of newly diagnosed patients with NSCLC. Use of PET-CT has been demonstrated to increase compliance with stage appropriate treatment and result in a decreased 5-year mortality (164). When used in conjunction with invasive mediastinal staging techniques, FDG-PET has a high sensitivity and specificity for mediastinal lymph node staging, allowing for improved target delineation when designing radiation treatment plans. By treating only involved mediastinal lymph nodes and avoiding traditional elective lymph nodes, PET-CT radiation planning technique has demonstrated at least equivalent local-regional control rate with decreased pneumonitis (165–167). Current studies are examining the role of mid-treatment FDG PET-guided imaging to guide treatment intensification (168).

Different metabolic pathways are currently under investigation for improving NSCLC prognostication and prediction. As discussed above, tumor hypoxia is an area of radioresistance within a tumor. Metabolic hypoxia PET tracers are being studied to guide radiation intensification. The most commonly used hypoxia markers are the nitroimidazole class of compounds with (18)F-fluoromisonidazole ((18)FMISO-) (169, 170) being the most studied. When using both FDG-PET and (18)F-FMISO imaging, a combined pattern can predict for risk of recurrence after SBRT (171). In head and neck cancer (18), F-FMISO PET/CT has been used to identify tumor areas with hypoxia requiring dose escalation (172).

Additionally, over the last decade, MRI of hyperpolarized 13C pyruvate - lactate conversion has matured in a technology which can be used to identify high risk solid tumors in early stage clinical trials (173, 174). In preclinical studies, 13C pyruvate HP-MRI has been advanced as a near real-time readout of intra-tumoral shifts in oxidative stress, and corresponding changes in carbon flux from pyruvate into lactate are able to be determined. At a basic level, HP-MRI leverages all of the aspects of altered solid tumor metabolism outlined above and may provide the first real-time biomarker of chemotherapy and radiation response in solid tumors. This may be more challenging to implement in lung cancer based upon motion artifact, but, along with CT-based imaging with dedicated radiotracers, represents an interesting avenue to explore (175–177).

## Discussion

Cancer cells have significant changes in glucose transport and metabolism, glycolysis, catabolic metabolism, mitochondrial oxidative cycle and aerobic fermentation, and the cancer TME. A better understanding of these complex interactions can help developing cancer therapeutics to improve chemoradiation and immunotherapy in patients with NSCLC. In this subset of locally advanced patients requiring chemoradiation, the prognosis has improved significantly with the addition of immunotherapy, but it still remains poor.

Metformin generated significant interest because initial retrospective data in diabetic patients suggested that metformin improved outcomes in patients receiving chemoradiation. Unfortunately, recent phase II clinical trials in nondiabetic patients did not show benefit of the addition of metformin. Taken together, these results demonstrate that in unselected nondiabetic patient populations, metformin does not improve outcomes with chemoradiation. So, how do we explain these findings and where does this leave metformin in NSCLC? Due to metformin being first-line treatment for type II diabetes mellitus, retrospective analysis could possibly be confounded in patients receiving appropriate management or patients taking metformin have less severe disease. As discussed previously, obesity is associated with type II diabetes mellitus, and the possibility that the previously discussed obesity paradox impacted patient outcomes instead of metformin cannot be excluded. Also, the hyperglycemia and hyperinsulinemia state in diabetic patients could cause differential effect on tumors such as the known increase in AMPK. The question if metformin will be useful in any clinical situations will likely depend on patient selection. As discussed previously, metformin may work better in certain patient populations such as *KRAS* and *STK11* mutations, suggesting there may be certain genetic mutations that would benefit. However, more preclinical and clinical data will be needed to confirm this. It remains to be seen how metabolic modulators can be used improve immunotherapy outcomes. Could metformin be added to adjuvant immunotherapy after chemoradiation to improve outcomes? Does the concurrent and adjuvant timing matter? Now that adjuvant immunotherapy is the standard of care, this question will become more important.

Incorporation of future metabolic modulators into chemoradiation therapy is not ready for prime time. Further work-up will be needed before incorporation of many of these metabolic modulators into clinical trials. However, there is rationale for incorporation in chemoradiation and immunotherapy which deserves significant consideration.

## Author contributions

AD and PP contributed equally to this manuscript. HS, AD, and PP were responsible for conceptualization, methodology, original draft and visualization. VS was responsible for conceptualization and reviewing/editing. HS is responsible for supervision. All authors contributed to the article and approved the submitted version.
